# The effectiveness of interventions to treat obesity in survivors of childhood brain tumors: a systematic review protocol

**DOI:** 10.1186/s13643-016-0274-9

**Published:** 2016-06-14

**Authors:** Kuan-Wen Wang, Marlie Valencia, Laura Banfield, Ruth Chau, Adam Fleming, Sheila K. Singh, Sarah Burrow, Russell J. de Souza, Lehana Thabane, M. Constantine Samaan

**Affiliations:** Department of Pediatrics, McMaster University, 1280 Main Street West, HSC-3A57, Hamilton, Ontario L8S 4K1 Canada; Division of Pediatric Endocrinology, McMaster Children’s Hospital, 1280 Main Street West, HSC-3A57, Hamilton, Ontario L8S 4K1 Canada; Health Sciences Library, McMaster University, Hamilton, Ontario Canada; Division of Pediatric Hematology/Oncology, McMaster Children’s Hospital, Hamilton, Ontario Canada; Division of Neurosurgery, Department of Surgery, McMaster Children’s Hospital, Hamilton, Ontario Canada; McMaster Stem Cell and Cancer Research Institute, McMaster University, Hamilton, Ontario Canada; Division of Orthopedic Surgery, Department of Surgery, McMaster University Medical Centre, Hamilton, Ontario Canada; Department of Clinical Epidemiology and Biostatistics, McMaster University, Hamilton, Ontario Canada; Department of Anesthesia, McMaster University, Hamilton, Ontario Canada; Centre for Evaluation of Medicines, St. Joseph’s Healthcare Hamilton, Hamilton, Ontario Canada; Biostatistics Unit, St Joseph’s Healthcare Hamilton, Hamilton, Ontario Canada

**Keywords:** Systematic review, Protocol, Obesity, Intervention, Children, Brain tumor, Brain tumor survivors, Cancer survivorship

## Abstract

**Background:**

Pediatric brain tumors are the most common solid tumors in children. Advances in understanding the hallmarks of cancer biology and novel therapies have led to an increasing number of survivors of childhood brain tumors (SCBT). However, these survivors are at an increased risk of obesity and cardiometabolic disorders that affect their quality of life and lifespan. It is important to define effective strategies to treat and prevent obesity in this population. This systematic review aims to investigate the effectiveness of lifestyle interventions, pharmacotherapy, and bariatric surgery on treating obesity in SCBT.

**Methods:**

Searches will be conducted in PubMed, MEDLINE, EMBASE, PsycINFO, SPORTDiscus, CINAHL, Cochrane Database of Systematic Review, Cochrane Central Register of Controlled Trials (CENTRAL), and Database of Abstracts of Reviews of Effect (DARE). In addition, ClinicalTrials.gov and ProQuest Dissertations and Theses A&I will be searched to identify relevant gray literature. The reference lists of eligible articles will be searched for additional studies. All screening, quality assessment, and data abstraction will be done independently by two reviewers. We will perform meta-analysis if there are sufficient studies.

**Discussion:**

This review will summarize evidence for the effectiveness of interventions used to reduce obesity risk in SCBT. This has significant implications for SCBT, as it can identify gaps in knowledge and provide insights into the development of new interventions to manage obesity in survivors, which may improve their outcomes.

**Systematic review registration:**

PROSPERO CRD42015025909

**Electronic supplementary material:**

The online version of this article (doi:10.1186/s13643-016-0274-9) contains supplementary material, which is available to authorized users.

## Introduction

Brain tumors are the most common solid tumors in children and constitute up to 20 % of childhood cancers [1]. Significant breakthroughs in understanding the hallmarks of cancer biology, coupled with advances in diagnostic imaging and improved therapies, have enhanced the survival rates of these children [2, 3].

As the number of survivors of childhood brain tumors (SCBT) increased, it has become apparent that survivors remain at risk of premature mortality [4–6] and the development of multiple comorbidities [7, 8]. Many SCBT develop chronic health conditions within years of their initial diagnosis [9], and one such morbidity is obesity [10–13]. In one study, obesity was reported in 36.5 % of SCBT, compared to 29 % in the general population [14, 15]. In the general population, the annual healthcare expenditures of obese individuals are about US$1360 higher than for their non-obese counterparts [16], and this is likely to be replicated in SCBT.

Addressing obesity in SCBT is crucial, as it increases the risk of cardiometabolic disorders in a similar fashion to the general population, and may contribute to premature mortality [17, 18]. Obesity is an independent risk factor for decreased survival in some children with brain tumors [19]. Understanding the drivers of obesity in SCBT will allow the development of precision-based strategies for reducing the risk of obesity and its cardiometabolic comorbidities, which in turn may improve the quality of life and lifespan of SCBT.

Obesity in SCBT is multifactorial and can be related to altered energy intake [20, 21], reduced mobility and physical activity [22–25], hypothalamic-pituitary damage [11], pituitary hormone deficiencies [26], sleep problems [27], vision problems, imbalance and pain [8, 28], mental health issues, and medications, e.g., antidepressants [29].

As obese children are likely to become obese adults [30–34], it is important to develop effective interventions to manage obesity from an early age. The purpose of this systematic review is to evaluate current evidence of effectiveness of interventions to manage obesity in SCBT.

### Research question

In survivors of childhood brain tumors, are the current interventions including lifestyle intervention, pharmacotherapy, and bariatric surgery effective in managing obesity?

### Study objectives

1. Measure the effectiveness of lifestyle interventions, pharmacotherapy, and bariatric surgery in the treatment of obesity in SCBT
2. Conduct a meta-analysis of primary studies, if appropriate, to gain a more precise estimate of the effectiveness of different strategies in managing obesity
3. Critically appraise existing evidence and identify gaps in the literature to provide future research directions

## Methods

The protocol for this systematic review is developed and reported with guidance from the Preferred Reporting Items for Systematic Review and Meta-Analysis-Protocols (PRISMA-P) statement (Additional file 1) [35].

### Eligibility criteria

This review will include studies involving boys and girls who are overweight or obese (BMI z-score ≥85th percentile) [36], with a diagnosis of brain tumor made under the age of 18 years. Randomized controlled trials (RCTs), quasi-RCTs, prospective or retrospective cohort studies, case-control studies, cross-sectional studies, and controlled or uncontrolled studies with before-and-after comparisons will be included [37].

There will be no restriction to the language or timing of publication. Conference proceedings, congress reports, and editorials will be hand searched for suggested relevant studies. We will exclude interim analyses, case reports, and pilot studies.

In studies where SCBT are included in an intervention with other cancer types, we will extract data for the brain tumor subgroup. If the data from subgroups are not published or pooled with data from survivors of other cancers, we will attempt to contact the authors to obtain the subgroup data.

The interventions included in the study are

- Lifestyle intervention: any form of modifications in subjects’ daily life including their dietary patterns, physical activity, and eating behaviors
- Pharmacotherapy: any administration of medications
- Bariatric surgery: any surgical approach performed with the intention of treating obesity, including adjustable gastric banding, sleeve gastrectomy, biliopancreatic diversion with duodenal switch, and gastric bypass

Studies that are entered into the databases up to February 1, 2016, will be screened for eligibility. The search will be updated to capture recently published literature.

### Outcome measures

#### Primary outcome

The primary outcome in this review is BMI z-score change from baseline to the end of the intervention and/or at follow-up.

#### Secondary outcomes

Secondary outcomes include changes in waist and hip circumference, waist-to-hip ratio, waist-to-height ratio, body fat percentage, and blood pressure as reported. We will also report changes in diabetes status, insulin resistance, and non-alcoholic fatty liver disease, if available. In addition, we will document changes in lipid levels including high-density lipoprotein, low-density lipoprotein, cholesterol, and triglycerides, if reported.

We will also abstract any adverse events observed during the study. Adverse events directly related to lifestyle interventions include back and shoulder pain, musculoskeletal injuries, and others [38, 39]. Adverse events for the pharmacological agents include insomnia, headaches, hypertension, and others [40]. Adverse outcomes for bariatric surgery include surgical complications, perioperative outcomes, and mortality as defined previously [41]. Additional adverse events will be included as reported.

### Search strategy

We will consult a Health Sciences librarian with expertise in systematic reviews when designing the search strategy. A proposed search strategy for MEDLINE is described in Table 1. Searches will be conducted in PubMed, MEDLINE, EMBASE, PsycINFO, SPORTDiscus, CINAHL, Cochrane Database of Systematic Review, Cochrane Central Register of Controlled Trials (CENTRAL), and Database of Abstracts of Reviews of Effect (DARE). We will search ClinicalTrials.gov and ProQuest Dissertations and Theses A&I to identify relevant gray literature. We will also search the reference lists of articles deemed eligible for inclusion in the analysis for relevant studies.

**Table 1:** Search strategy for MEDLINE

1	exp Child/
2	child*.mp.
3	p?ediatric*.mp.
4	exp Adolescent/
5	adolescen*.mp.
6	youth*.mp.
7	exp Adult/
8	adult*.mp.
9	Young Adult/
10	1 or 2 or 3 or 4 or 5 or 6 or 7 or 8 or 9
11	exp Brain Neoplasms/
12	exp Cranial Nerve Neoplasms/
13	exp Neuroectodermal Tumors/
14	cerebroma*.mp.
15	exp Glioma/
16	glioma*.mp.
17	astrocytoma*.mp.
18	oligoastrocytoma*.mp.
19	astroglioma*.mp.
20	glioblastoma*.mp.
21	retinoblastoma*.mp.
22	pinealoma*.mp.
23	pineoblastoma*.mp.
24	pinealoblastoma*.mp.
25	pinealblastoma*.mp.
26	pineal blastoma*.mp.
27	pineocytoma*.mp.
28	pinealocytoma*.mp.
29	craniopharyngioma*.mp.
30	ependymoma*.mp.
31	subependymoma*.mp.
32	ependymoblastoma*.mp.
33	ganglioglioma*.mp.
34	gliosarcoma*.mp.
35	medulloblastoma*.mp.
36	exp Germinoma/
37	germinoma*.mp.
38	Meningioma/
39	meningioma*.mp.
40	oligodendroglioma*.mp.
41	exp Neurofibromatoses/
42	neurofibromatos*.mp.
43	PNET*.mp.
44	neurocytoma*.mp.
45	choroid plexus papilloma*.mp.
46	exp Neoplasms/
47	cancer*.mp.
48	tumo?r*.mp.
49	neoplasm*.mp.
50	11 or 12 or 13 or 14 or 15 or 16 or 17 or 18 or 19 or 20 or 21 or 22 or 23 or 24 or 25 or 26 or 27 or 28 or 29 or 30 or 31 or 32 or 33 or 34 or 35 or 36 or 37 or 38 or 39 or 40 or 41 or 42 or 43 or 44 or 45 or 46 or 47 or 48 or 49
51	exp Obesity/
52	obes*.mp.
53	Overweight/
54	over weight.mp.
55	overweight.mp.
56	51 or 52 or 53 or 54 or 55
57	life style*.mp.
58	lifestyle*.mp.
59	exp Diet/
60	diet*.mp.
61	exp Nutrition Therapy/
62	nutrition.mp.
63	behavi*.mp.
64	exp Exercise Therapy/
65	kinesiotherap*.mp.
66	physical activ*.mp.
67	exp Exercise/
68	exercis*.mp.
69	walk*.mp.
70	jog*.mp.
71	run*.mp.
72	swim*.mp.
73	exp Bariatrics/
74	bariatric*.mp.
75	bariatric surger*.mp.
76	gastrojejunostomy.mp.
77	gastric bypass.mp.
78	stomach bypass.mp.
79	jejunoileal bypass.mp.
80	lipectomy.mp.
81	gastroplasty.mp.
82	stomach stapling.mp.
83	drug*.mp.
84	pharm*.mp.
85	exp Weight Reduction Programs/
86	((weight reduc* or weight los*) adj5 surger*).mp.
87	((weight reduc* or weight los*) adj5 program*).mp.
88	57 or 58 or 59 or 60 or 61 or 62 or 63 or 64 or 65 or 66 or 67 or 68 or 69 or 70 or 71 or 72 or 73 or 74 or 75 or 76 or 77 or 78 or 79 or 80 or 81 or 82 or 83 or 84 or 85 or 86 or 87
89	Body Weight/
90	body mass*.mp.
91	BMI.mp.
92	exp Body Weight Changes/
93	exp Body Weights and Measures/
94	body fat.mp.
95	waist-height ratio*.mp.
96	waist to height ratio*.mp.
97	adipos*.mp.
98	body size*.mp.
99	waist circumference*.mp.
100	hip circumference*.mp.
101	weight*.mp.
102	height*.mp.
103	waist-hip ratio*.mp.
104	waist to hip ratio*.mp.
105	skinfold thickness*.mp.
106	89 or 90 or 91 or 92 or 93 or 94 or 95 or 96 or 97 or 98 or 99 or 100 or 101 or 102 or 103 or 104 or 105
107	10 and 50 and 56 and 88 and 106

### Data management

Two independent reviewers will perform data abstraction and quality assessment. Disagreement between the two reviewers will be resolved by discussion, with subsequent involvement of a third reviewer to arbitrate disagreements. Excel spreadsheets will be used to manage study records during the screening process. We will use the Grading of Recommendations Assessment, Development and Evaluation Profiler (GRADEpro) software to create tables for summary of findings and quality assessment [42].

### Data screening

**Fig. 1:**
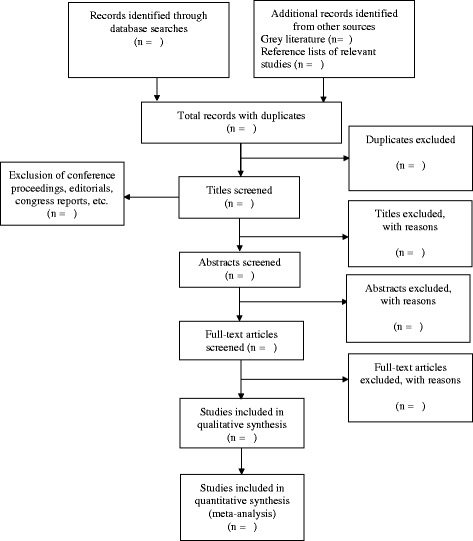
Flow diagram of the article screening process

Duplicates will be removed, followed by screening of titles and abstracts. Full-text articles that meet the inclusion criteria will be retrieved and screened. Screening at all steps will be conducted independently by two reviewers, who will meet after each step to ensure consistency and to resolve conflicts. In the case of persisting disagreement, a third reviewer will be consulted. A flow diagram will be included to report the screening process (Fig. 1) [43, 44].

### Data abstraction

Data will be extracted independently by two reviewers, using a data abstraction form specifically designed for this systematic review. Details to be collected include title, authors, publication date, journal name, setting, country, funding source, study design, study duration, eligibility criteria, sample size, and methods used for brain tumor diagnosis including imaging, histology, and clinical assessment.

Participants’ characteristics include age at diagnosis of brain tumor and at study enrollment, sex, ethnicity, and brain tumor location and laterality. Treatment details include radiotherapy type (fractionated or non-fractionated) and dose, chemotherapy type, dose and duration, and surgery details (total resection, partial resection, shunting, ventriculostomy, others).

Detailed description of the obesity interventions will be recorded including study design, components, duration, and adverse events. We will document primary and secondary outcomes of the studies. Adjustment for confounders and details of the statistical analyses performed will be extracted as well as study results. We will attempt to retrieve incomplete data by contacting the corresponding authors of published work.

### Quality assessment

The Risk of Bias Assessment Tool from the Cochrane Collaboration will be used to assess RCT [45]. This tool includes six domains: sequence generation, allocation concealment, blinding, incomplete data, selective reporting outcomes, and other sources of bias. Each RCT will be rated as having either a high, low, or unclear risk of bias.

The Risk of Bias In Non-randomized Studies—of Interventions (ROBINS-I) assessment tool will be used for non-randomized studies such as cohort studies [46]. This tool includes three domains: pre-intervention, at-intervention, and post-intervention.

In the pre-intervention domain, bias due to confounding and participant selection are evaluated. Possible confounding factors include brain tumor location, type, treatments, years of survival, age, sex, pubertal stage, baseline body composition, and presence of comorbidities such as metabolic syndrome and hormonal deficiency. Bias due to misclassification of the intervention status is assessed in the at-intervention domain. The post-intervention domain includes bias due to departures from the intended interventions, missing data, methods of outcome measurements, and selective reporting outcomes. In particular, co-interventions between lifestyle interventions, pharmacotherapy, and bariatric surgery can contribute to bias during the post-intervention domain. For example, participants may take antiobesity agents while they are on diet restriction. Each non-randomized study will be rated as having either a low, moderate, serious, critical, or unclear risk of bias.

The quality of uncontrolled studies will be assessed with a checklist developed by the University of Alberta Evidence-based Practice Center (UAEPC) [47]. This checklist evaluates selection bias, incomplete data, and the methods of outcome assessments. We will tabulate risk of bias for all included studies and discuss its impact on the meta-analysis.

The quality of evidence will be assessed using the Grading quality of evidence and strength of recommendations (GRADE) guidelines [48]. The GRADE guideline covers risk of bias, inconsistency, indirectness, imprecision, and publication bias. The overall quality of evidence is reported by each outcome measure as high, moderate, low, or very low.

### Data analysis

Detailed characteristics of the included studies will be provided, in addition to a meta-analysis if applicable. We will analyze each intervention separately, and outcomes will be analyzed separately based on study designs. We will perform a meta-analysis if two or more studies are identified per intervention.

Dichotomous outcomes will be reported as odds ratio, while continuous outcomes will be reported as standardized mean differences and 95 % confidence intervals. Expecting high levels of heterogeneity, our primary approach will emphasize the random effects estimate if more than ten studies can be identified [49]. Otherwise, both random effect and fixed effect models will be presented.

Inconsistency index (*I*^2^) and *P* values will be used to quantify heterogeneity. The interpretation of the *I*^2^ will be based on the threshold set by the Cochrane Collaboration [50]. If appropriate, a stratified analysis by sex will be pursued to identify a source of heterogeneity, as female SCBT are more at risk of developing obesity than males [8, 10].

If sufficient studies are identified for an outcome (≥10), we will perform sensitivity analysis by excluding outlier, small-sized, or highly biased studies to determine the impact of these studies on the meta-analysis result. To investigate publication bias, we will create a contour-enhanced funnel plot and use Egger’s test and visual inspection to determine plot asymmetry, if there are ten or more studies for an outcome [51].

All meta-analyses will be conducted using Review Manager software version 5.3 (RevMan 5.3) [52] while Comprehensive Meta-Analysis software version 3 (CMA 3.0) will be used for Egger’s test [53]. When meta-analysis is not appropriate, a table for summary of findings will be created using GRADEpro software and a narrative summary will be reported. The results of this systematic review will be presented according to the Preferred Reporting Items for Systematic Reviews and Meta-Analyses (PRISMA) guidelines [43, 44]. When amendments of the protocol are needed, we will document the date and the rationale for these changes.

## Discussion

As the number of SCBT increased over time, it has become apparent that the burden of surviving a brain tumor is significant [4, 6, 12, 13]. Obesity is a critical comorbidity to address in survivors, as it drives the risk of cardiovascular diseases, type 2 diabetes, metabolic syndrome, and hypertension [7, 8, 17–19]. This reduces the quality of life and lifespan of the survivors and increases healthcare system utilization.

In order to improve health outcomes in SCBT, it is important to develop evidence-based interventions to treat and prevent obesity and its cardiometabolic comorbidities.

The findings from this systematic review will have important implications for SCBT, as it will provide insights into the current best form of obesity intervention for these patients. The review will also define gaps in knowledge and help improve the quality of life and lifespan of SCBT by guiding the design of new interventions to target obesity and its cardiometabolic comorbidities.

## Abbreviations

BMI, body mass index; CENTRAL, Cochrane Central Register of Controlled Trials; CINAHL, Cumulative Index to Nursing and Allied Health Literature; CMA 3.0, Comprehensive Meta-Analysis version 3; DARE, Database of Abstracts of Reviews of Effect; EMBASE, Excerpta Medica Database; GRADE, Grading of Recommendations Assessment, Development and Evaluation; PRISMA, Preferred Reporting Items for Systematic Review and Meta-Analyses; RCT, randomized controlled trial; RevMan 5.3, Review Manager software version 5.3; ROBINS-I, Risk Of Bias in Non-randomized Studies—of Interventions; SCBT, survivors of childhood brain tumors; UAEPC, University of Alberta Evidence-based Practice Center

## Additional file

Additional file 1: PRISMA-P checklist. This checklist includes recommended items to address in a systematic reviews protocol and their location in this protocol. (DOCX 40 kb)
